# Improvement in Stress, General Self-Efficacy, and Health Related Quality of Life following Patient Education for Patients with Neuroendocrine Tumors: A Pilot Study

**DOI:** 10.1155/2013/695820

**Published:** 2013-04-23

**Authors:** Trude Haugland, Marijke Veenstra, Morten H. Vatn, Astrid K. Wahl

**Affiliations:** ^1^Clinic for Cancer, Surgery and Transplantation, Oslo University Hospital, Rikshospitalet, 0424 Oslo, Norway; ^2^Department of Nursing Science, University of Oslo, 0318 Oslo, Norway; ^3^Faculty of Medicine, Department of Public Health and Primary Health Care, University of Bergen, 5020 Bergen, Norway; ^4^NOVA-Norwegian Social Research, 0208 Oslo, Norway; ^5^Epi-Gen-Institute, Campus Ahus, Akershus University Hospital, 1478 Lorenskog/Faculty of Medicine, University of Oslo, 0318 Oslo, Norway; ^6^Department of Health Sciences, University of Oslo, 0317 Oslo, Norway

## Abstract

The purpose of the study was to evaluate changes in general self-efficacy, health related quality of life (HRQoL), and stress among patients with neuroendocrine tumors (NET) following a multidisciplinary educational intervention. Forty-one patients were enrolled in this exploratory pilot study. A total of 37 patients completed the full 26-week intervention based on the principles of self-efficacy. General self-efficacy was measured by the General Self-Efficacy Scale, HRQoL was measured with the SF-36, and stress was measured with the Impact of Event Scale. Mixed effect models were used to evaluate changes in general self-efficacy, mental and physical components of HRQoL, and stress adjusting for demographic and clinical variables. Results showed significant improvements in patients' general self-efficacy (*β* = 0.71; *P* < 0.05), physical component scores of HRQoL (*β* = 3.09; *P* < 0.01), and stress (*β* = −2.10, *P* = 0.008). Findings suggest that patients with NET have the capacity to improve their ability to cope with their disease, problem-solve, improve their physical status, and reduce their stress following an educational intervention based on the principles of self-efficacy. These preliminary data provide a basis for future randomized controlled trials to test interventions to improve HRQoL for patients with NET.

## 1. Introduction

The incidence of the relatively slow growing and rare types of neuroendocrine tumors (NET) is 5.3/100,000, and the prevalence is 35/100,000 [1]. Neuroendocrine cells are distributed widely throughout the body, including the nervous and endocrine systems. Neuroendocrine tumors produce and secrete regulatory hormones, giving rise to symptoms including fatigue, flushing, diarrhea, food intolerance, restlessness, dyspnea, fluctuations in mood [2], and pain [3].

Symptoms vary widely and may occur late in the course of the disease, depending on the type of hormone affected and the rate of secretion and localization, thus making diagnosis challenging. In the majority of cases, a definitive diagnosis is not made until after the tumor has metastasized [4]. Thus, NET represents a clinical challenge in diagnosis, treatment, and care. Palliative treatment includes biological agents, such as somatostatin analogues, interferon, and embolization of liver metastases, and frequently gives rise to side effects that may be similar to the symptoms of NET [5, 6].

Stress is a common reaction to cancer [7–11] and may influence patients' adaptation to the disease [5]. Consequently, stress can have a sustained impact on patients' ability to function, which in turn may increase the risk of reduced health related quality of life (HRQoL) [7–9, 12–16]. Patients with NET have demonstrated decreased HRQoL in previous studies [17–21]. Interventions enabling patients to cope with stress and thus improving their HRQoL may be a complementary treatment option. Norwegian legislation [22] mandates that patients with chronic diseases have access to information and education that contribute to coping and maintenance of independence and functional ability. Bodenheimer et al. have developed the Chronic Care Model to guide interventions aimed at improving chronic illness management [23]. The model includes elements such as self-management support, which involves collaboratively helping patients and their families acquire skills and confidence to manage their chronic illness, providing self-management tools and routinely assessing problems and accomplishments. Core components of chronic care interventions could also be based on the principles of self-efficacy to help motivate patients to self-manage their symptoms and adopt new skills and competencies. Different components of patient education such as patient and family knowledge, provision of emotional and psychosocial support, self-efficacy, coping skills, and relaxation training have been shown to be effective in reducing stress and improving HRQoL across settings and disease conditions [13, 15, 16, 24–28].

Self-management interventions in general, and disease-specific interventions in particular, have shown varying levels of efficacy on outcomes such as physical health [29–31], physical function [32], health status [33], general health [34], disease-specific self-efficacy [35, 36], weight self-efficacy [31], and perceived stress [29]. However, these studies may not be comparable because of theoretical and methodological differences. No studies have reported on educational interventions based on self-efficacy in patients with NET. Hence, the purpose of the study was to evaluate changes in stress, general self-efficacy, and HRQoL among patients with NET, following an educational intervention based on the principles of self-efficacy.

## 2. Methods

### 2.1. Design and Sample

**Figure 1:**
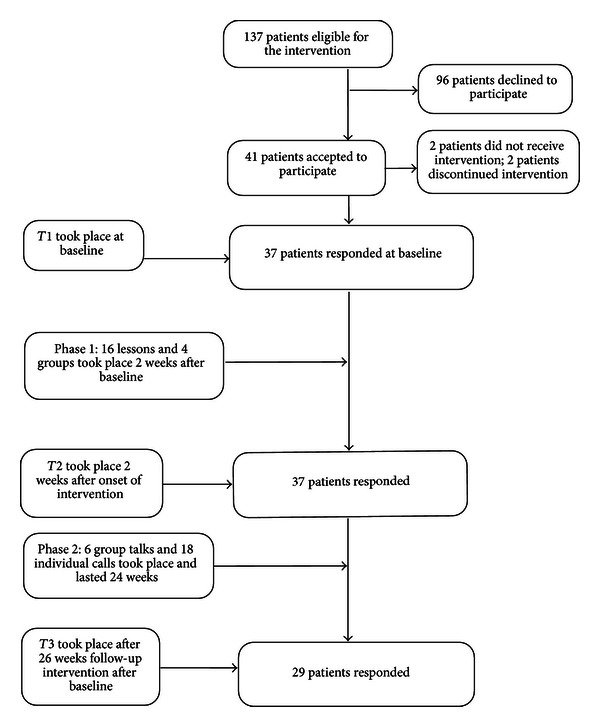
Flow diagram for the participants of the intervention and the time of test points.

A single-group pre/posttest design was used. Measures were completed at three time points (T1–T3): baseline, following phase 1 of the intervention (2 weeks), and at the completion of the intervention (26 weeks). All 137 patients referred to three of the five Norwegian regional university hospital NET centers (Bergen, Trondheim, and Oslo) from September 2005 to December 2007 were invited to participate. Inclusion criteria were ≥18 years of age, NET diagnosis within the last 24 months, undergoing medical treatment for NET, having tumors restricted to the gastrointestinal tract, and able to speak and read Norwegian. Those who had previously completed an educational program, were terminally ill, had undergone radical surgery, or were suffering from cognitive or mental deficits were excluded. The study was approved by the Regional Ethics Committee in Health, Region II (South) of Norway and the Norwegian Social Science Data Services. Written informed consent was obtained. Patients received questionnaires by mail following enrollment. Those who did not return the questionnaire within two weeks received a reminder letter by mail. Forty-one patients agreed to participate (response rate 30%), 37 returned the pretest questionnaire two weeks prior to the start of the intervention (90%), and 29 (71%) patients returned the questionnaire on completion of the 26-week intervention program (see Figure 1).

### 2.2. The 26-Week Intervention

 Bandura's Social Cognitive Theory focusing on self-efficacy has been the foundation for successful interventions in previous research [37]. 

Self-efficacy is the belief in one's competence to take on difficult or novel tasks and to cope with adversity arising from demanding situations [38]. Self-efficacy relates to actions and control during specific situations and is outcome specific. Self-efficacy is commonly understood as domain specific, in that one can have more or less firm self-beliefs in different domains or particular situations of functioning. 

A general sense of self-efficacy, however, refers to a global confidence in one's coping ability across a wide range of demanding or novel situations and reflects a person's general problem-solving ability. General self-efficacy aims at a broad and stable sense of personal competence to deal effectively with a variety of stressful situations over time [38]. Improving a patient's general self-efficacy may act as a basis for problem-solving strategies. 

General strategies for enhancing self-efficacy incorporate the following: (1) mastery experience, which includes giving positive feedback when patients have performed a desired activity; (2) vicarious experience, or modeling others, gained through watching someone else in a similar situation who has had success in performing activities; (3) verbal persuasion, which involves encouraging patients to believe that they have the ability to achieve their goals; and (4) strengthening physical and psychological states, which includes teaching patients about disease-specific knowledge and psychological reactions to severe illness [39]. The multidisciplinary intervention consisted of 10 sessions over a period of 26 weeks. The program included lectures, group discussions, and individual telephone calls in two phases (Figure 1). The intervention integrated both domain-specific and general problem solving, such as dealing with symptoms and other peoples' reactions to severe disease, respectively. 

#### 2.2.1. Phase 1

 The first phase took place over four consecutive days and included lectures and group discussions. Beforehand, all patients participated in a 45-minute orientation session to introduce them to the study protocols and goals and the principles of general self-efficacy. This was followed by an introductory session where the patients received a booklet containing the intervention protocol. Lectures consisted of one 45-minute didactic session on each of the four mornings followed by a 60-minute group discussion in the afternoon. 

The goals of the morning sessions were to improve the patients' knowledge of NET, side effects of medication and treatment, and what to expect in follow-up sessions. In the afternoon group discussions, the patients participated in motivational training in self-efficacy enhancing strategies led by the study nurse. Motivational training included training in mastery techniques by empathetic guidance in goal setting, writing goals, and discussing how best to achieve them based on previous successful experiences. By specific and constructive questioning on patient priorities the participants were taught how to break down large goals into smaller, weekly action plans that were measurable, realistic, and attainable. Vicarious learning was modeled on other patients' successful self-care activities by encouraging patients to share positive self-care solutions within the group. The nurse also taught patients how to mutually encourage each other in self-efficacy thoughts as well as supporting constructive alternatives in setting goals if barriers were encountered. One strategy used to strengthen patients' physical and physiological status was to improve their knowledge about NET and mental reactions on severe disease. In addition, learning about the positive outcomes of physical activity on health helped patients understand that participation in physical activities was safe and beneficial. Patients also learned about the trajectory of their illness and how best to respond to symptoms and when it was appropriate to contact their physician or nurse. 

#### 2.2.2. Phase 2

 The second phase of the study lasted for 24 weeks and included six follow-up group discussions and 18 individual telephone calls, all conducted by six nurses specializing in NET. Patients met at their regional hospital for 90-minute group discussions every four weeks. Four to six individuals took part in each group discussion. Weekly telephone calls (45 minutes) were made between group meetings. The nurses led motivation and training exercises to assist the patients in achieving their goals by giving positive feedback and focusing on individual strengths and successes (Table 1).

To ensure adherence to the intervention, the principal investigator trained the study nurses on the application of self-efficacy principles during three 45-minutes sessions and in additional booster sessions. Nurses also received a training booklet to refer to as needed. A critical self-evaluation was performed after every group session, by reflecting on how the four principles of self-efficacy had been discussed and supported. Reflections were then logged for follow-up discussion with the principal investigator.

## 3. Measures

### 3.1. Background Characteristics

 Sociodemographic variables measured were gender, age, education level, marital status (living with partner or not), employment status (employed or not), and income (measured in Norwegian Kroner). Disease-specific symptoms were listed, and patients were asked to indicate how often they experienced each of them, measured as never (0), occasionally (1), often (2), very often (3), and most of the time (4). Comorbid conditions were measured dichotomously (yes/no).

### 3.2. General Perceived Self-Efficacy

 The General Perceived Self-Efficacy Scale is a 10-item scale that measures general self-efficacy. Each item is scored from 1 (not at all true) to 4 (completely true). The summary score ranges from 10 to 40, with the highest score indicating high self-efficacy. The scale has demonstrated validity and reliability across cultures [40]. Mean substitution was used to calculate the score when fewer than 50% of item scores were missing.

### 3.3. Health Related Quality of Life

 Health related quality of life was measured by the SF-36. The eight SF-36 subscales were transformed into standardized physical (PCS) and mental (MCS) component scores [41], using normative United States (US) data. Both PCS and MCS component scores were analyzed. A deviation of 10 points from the mean score (50) represents a difference of one standard deviation in the general US population. The scales and items of the SF-36 have shown satisfactory reliability, validity, and responsiveness to changes in health status [42] across a broad range of patient populations [43]. Normative values for the SF-36 have been published for the Norwegian population [43]. Mean substitution was used to calculate the score for dimensions when fewer than 50% of the scores were missing, as suggested in the SF-36 manual [44].

### 3.4. Impact of Event Scale

 A modified version of the Impact of Event Scale (IES) was used to measure current stress specifically related to NET. Seven items assess intrusive thoughts, which can be described as invasive ideas, images, feelings, or bad dreams about cancer. Eight items assess avoidance behaviors, which are described as consciously recognized avoidance of certain ideas, feelings, or situations. Each item is scored on a 6-point scale from 0 (never) to 5 (often). The total score ranges from 0 to 75. Higher scores indicate higher stress. The IES questionnaire has been found to be valid and reliable [45]. All patients except one answered all items on the scale. Mean substitution was calculated (mean of 7 items) for the three items that were missing for this person.

### 3.5. Statistical Analyses

 Descriptive statistics were used to assess participant characteristics (Table 2) and outcome measures (stress, general self-efficacy, PCS, and MCS) (Table 3). Mixed effect models with unstructured covariance were applied to evaluate overall change in the four outcome measures from T1 to T3 and change across individuals [46]. This analysis does not require list-wise deletion of missing data. These analyses enabled estimation of average within-patient change over time on our primary outcome measures and the rate of change across patients. We adjusted for age, gender, symptoms, and comorbidity in all analyses. For each of the explanatory variables, the effects of interaction with time were evaluated separately. Gender, baseline age, and comorbidity were held constant across time and were considered fixed predictors in the models. Changes in the number and frequency of symptoms over the three time points were included as time variables. Given the small sample size, we used time (T1, T2, and T3) as a linear variable. Because the average change in cancer-related stress deviated from linearity, we also ran the model using time as a categorical variable, which gave similar results.

Effect size (ES) for changes in stress, general self-efficacy, and HRQoL was calculated by computing the differences in mean scores divided by the pooled standard deviation to estimate and interpret clinically meaningful changes [47]. Effect sizes of 0.20 to 0.49 were regarded as small, of 0.50 to 0.80 as moderate, and greater than 0.80 as large [47].

## 4. Results

### 4.1. Background Characteristics

The demographic and clinical characteristics of the sample (*n* = 37) are described in Table 2. Of these, 37 completed the intervention (see Figure 1). Their ages ranged from 36 to 80 years, with almost equal numbers of women (*n* = 17) and men (*n* = 20). The majority were currently married or cohabitating (76%). The participants were relatively well educated, with 73% having a high school education or higher. In terms of employment, 35% were currently working, 56% were retired, and the remaining 9% did not respond to the question. The average income was high (542 000 NOK). Thirty-six patients (89%) had been diagnosed with NET more than 6 months before enrollment in the study (mean = 13 months). The most frequently reported symptom (89%) was food intolerance, and flushing and restlessness were the least reported symptoms (19%), while 22% had comorbid conditions such as arthritis, breast cancer, and myocardial infarction. A comparison of demographic and clinical characteristics between those who completed the intervention and those who withdrew showed no significant differences in age (*P* = 0.90) or gender (*P* = 0.51).

### 4.2. Changes in Stress, General Self-Efficacy, and Health Related Quality of Life

Mean scores and effect sizes for measures from T1 to T3 are shown in Table 3. The estimated ES was 0.16 for stress, 0.32 for general self-efficacy, and 0.37 for PCS. There was a statistically significant improvement in stress following the intervention (*β* = –2.10; *P* = 0.008) after adjusting for gender, age, comorbidities, and symptoms. Levels of stress were higher for those with more than one symptom (*β* = 7.96; *P* = 0.001). General self-efficacy showed a small but statistically significant improvement over time (*β* = 0.71; *P* < 0.05). The PCS component of HRQoL was significantly improved (*β* = 3.09; *P* < 0.01) after adjusting for gender, age, comorbidities, and symptoms. The presence of comorbid conditions and/or more than one symptom was associated with a lower PCS. There was an interaction between PCS and gender, with women showing less improvement in PCS than men (*β* = –2.77; *P* < 0.05) (Table 4).

## 5. Discussion

This is the first study focusing on the evaluation of an educational intervention based on the principles of self-efficacy in patients with NET. Patients participated in an extensive education program over 26 weeks that focused on problem-solving strategies in relation to living with the diagnosis of NET. Our study found significant improvements in general self-efficacy, physical HRQoL, and stress following the intervention. The improvements in PCS and general self-efficacy were small but may be of clinical importance. The clinical relevance of the improvement in stress is more uncertain. In social cognitive theory, the concept of self-efficacy represents a person's judgment about her/his ability to overcome barriers to achieve change [37]. Improved levels of general self-efficacy may indicate that a person has enhanced competence in problem-solving strategies and hence is able to tackle demanding situations such as stress.

NET is one of several cancer diagnose, and it is reasonable to use cancer studies in general as a comparison group for our results. Systematic reviews of intervention studies in which patients acquired new skills and gained confidence to manage their disease show various effects with regard to outcomes such as stress, self-efficacy, and HRQoL [13, 15, 16, 28]. For instance, findings from a literature review of randomized controlled trials (RCT) by McGregor and Antoni demonstrate reduced levels of stress following cognitive behavioral interventions in women treated for breast cancer [11], similar to results from the present study. In a RCT using individual psychosocial support based on techniques derived from cognitive behavioral therapy in women with breast cancer (*n* = 425), stress (measured by the intrusion subscale of the Impact of Event Scale) was reduced following the intervention [48]. In contrast, Chan et al. conducted a RCT that included a psychoeducational intervention in women with gynecologic malignancy (*n* = 155). The intervention was aimed at helping patients understand the link between thoughts, emotions, and physical well being. Findings revealed no improvements in stress as measured by the Impact of Event Scale [25].

With regard to self-efficacy and in contrast to the present study, Chan et al. also found no improvements in general self-efficacy following a psychoeducational intervention in women with gynecologic malignancy (*n* = 155) [25]. Tamagawa et al. [28] summarized the benefits from psychosocial interventions in oncology: the types of interventions were as follows: cognitive-behavioral stress management cognitive behavioral therapy; expressive writing, support group alone; psychoeducation, support group plus psychoeducation; psychoeducation and life style and coping training; individual supportive counseling; a booklet including self-management skills and stories of other patients' experiences; and a booklet for treatment decision making. The results indicated that those with low levels of self-efficacy initially benefited from psychosocial interventions, while those with higher levels of self-efficacy at baseline did not [28]. Similar to our findings, educational interventions have led to improvements in HRQoL in other cancer populations. In a RCT of two interventions, one based on education only, and the other based on education and group discussion, men (*n* = 279) with prostate cancer demonstrated significant improvements in PCS in the education-plus-discussion group [30]. 

Doorenbos et al. showed that physical function measured by the SF-36 improves significantly in individuals with solid tumor cancer (*n* = 237) following an intervention based on self-care management information, counseling and support, and problem-solving and communication skills derived from cognitive behavioral theory [32]. However, Chan et al. found no improvement in cancer-specific physical HRQoL following a psycho-educational and cognitive intervention in women with gynecologic malignancy (*n* = 155) [25].

In the present study, men had a significantly larger change in PCS than women. Men's lower baseline scores for PCS may explain this difference, because men had greater potential for improving their PCS. Consistent with earlier research [32], comorbid conditions were related to reduced physical function. Despite this finding, research has shown that interventions based on principles of self-efficacy have positive effects on those at greater risk of lower physical functioning [32].

Symptoms such as diarrhea or dyspnea may alter the physical activities of patients with NET. Consistent with research in individuals with solid tumor cancers [32] and men being treated for prostate cancer [49], findings from this study indicated that frequent symptoms were associated with lower PCS. It is possible that patients who experience symptoms more frequently may gain greater benefit from psychosocial interventions than those who are less troubled by symptoms [28].

Since self-efficacy is behavior specific and not “general,” the items included on the General Self-Efficacy Scale were reviewed. They relate to problem solving and problem management. The scale might more appropriately be named “Problem Management Self-Efficacy.” These are very important behaviors for people with chronic illnesses to learn and in which to become confident in doing. The education program developed for the patients in this study included content and skills on problem solving and problem management. Therefore, the positive changes in what is called “general self-efficacy” might be “problem management self-efficacy.” Since this is an ongoing discussion, we are mentioning it here to inform our readers. This was the tool we used in the study, and we did achieve significant improvement. Further research related to clarifying the underlying dimensions of the scale should be undertaken.

In the present study, the small sample size is a limitation. The majority of patients who were eligible for the study declined to participate. The fact that the patients were geographically dispersed also limited their participation in the intervention. The single group design was unavoidable because of the small number of identifiable patients with NET in Norway. However, the significant findings indicate that the sample was large enough to detect significance where it existed. Furthermore, due to the lack of control group spontaneous remission is an evident rival hypothesis for our findings. It can be expected that without any intervention, patients will adapt to their situation, stress will decrease, and their general self-efficacy and HRQoL will increase over time. Therefore, the assertions about what has been proven in this study should be verified in a satisfactory powered and controlled trial. Finally, the intervention was long and somewhat complicated, and it is not possible to determine if a more streamlined intervention would be efficacious. Web-based interventions may aid in overcoming this limitation, make it possible to include people living in rural areas, and may also be more cost effective.

Despite limitations, the study was feasible and demonstrated that patients with NET show improvements in functioning following a self-efficacy-based educational intervention. 

The findings have implications for oncology nursing. As the intervention was time consuming for the research team, it is possible that part of the intervention could be integrated into discharge education. Future research on stress, self-efficacy, and HRQoL in patients with NET may use cognitive theory as a basis for a psychosocial intervention. We demonstrated that using principles of self-efficacy to facilitate mastery of self-care activities and symptom management is feasible in patients with NET. Interventions directed towards problems caused by challenges that are specific to NET could be a focus of future research. Focusing on behavior-specific self-efficacy, NET-related stress, and NET-related HRQoL may add knowledge about specific problems in order to achieve reduced levels of stress, so that patients become confident in handling stressful situations and undertaking activities. In addition, an initial assessment of patients' social support and behavior could be used to identify and target vulnerable patients.

## 6. Conclusion

In this pilot study, we found decreased stress and enhanced general self-efficacy and PCS following the educational intervention in patients with NET. Learning about the disease and engagement in strategies to enhance self-efficacy may strengthen patients problem solving. Thus, the patients may have overcome barriers to achieve change and are able to tackle demanding situations such as stress. However, further research using experimental design is needed to evaluate effects of such an intervention.

## Figures and Tables

**Table 1:** Description of the 26-week intervention.

Intervention topic (numbers × minutes)	Content	Facilitated by
Phase 1 week 1		
Introduction (1 × 45)	(i) Study protocol, information about goal setting and information about principles of self-efficacy. The patients received a booklet with the intervention protocol to take home	(i) The principle investigator
Lectures (16 × 45)	(i) Disease-specific knowledge (ii) Psychological reactions on severe illness (iii) Social rights (iv) Physical activity (v) Nutrition	(i) Physician (ii) Psychologist (iii) Social worker (iv) Physiotherapist (v) Nutritionists
Group sessions (4 × 60)	*Discussions based on the principles of self-efficacy in order to enhance problem*-*solving strategies* (i) *Mastery experiences: *utilize previous, optimistic, and positive experiences and evaluate their written goals (ii) *Vicarious experiences: *utilize significant other's optimistic and positive experiences in the coping process (iii) *Verbal persuasion: *encouraged in believing that they could achieve their goals and in sharing experiences as well as supporting other's coping strategies (iv) *Strengthening physical and psychological state: *utilize disease-specific knowledge and enable the patients to recognize disease-specific symptoms and when to contact health care for assistance	(i) Nurse
Phase 2 week 2–6		
Group sessions (6 × 90)	(i) *Discussions based on the principles of self-efficacy in order to enhance problem*-*solving strategies *	(i) Nurse
Individual telephone calls (18 × 30)	(i) *Individual support based on the principles of self-efficacy*: follow up of weekly goals and reflective notes	(i) Nurse

**Table 2:** Sample characteristics at time of inclusion (*n* = 37).

Background variables	*N* (%)	Mean (range)
Age mean	30 (81)	60 (36–80)
Missing	7 (19)	
Income NOK	29 (78)	542^a^ (100^a^–4500^a^)
Missing	3 (8)	
Disease duration (months)	29 (78)	13 (1–24)
Missing	2 (5)	
Gender		
Male	17 (46)	
Female	20 (54)	
Marital status		
Single, divorced, widowed	9 (24)	
Married, cohabitation	28 (76)	
Education		
Primary < 10 years	10 (27)	
High school 10–13 years	11 (30)	
Secondary > 13 years	16 (43)	
Working situation		
Working	13 (35)	
Retired	21 (56)	
Missing	3 (9)	
Symptoms frequently		
Diarrhea	13 (35)	
Fatigue	13 (35)	
Nutrition intolerance	33 (89)	
Flushing	7 (19)	
Restlessness	7 (19)	
Fluctuating mood	11 (30)	
Others *n* (%)	3 (9)	
Comorbid conditions (≥1)	8 (22)	

NOK: Norwegian currency (kroner).

^a^: 000.

**Table 3:** Pretest and posttest mean scores, standard deviation (SD), and effect size for scores from T1 to T3 for mental and physical component scores, stress, and general self-efficacy in patients with neuroendocrine tumors (T1, *n* = 36-37; T2, *n* = 37; T3, *n* = 29-30).

	Pretest T1 Mean (SD) (*n* = 36)	Posttest T2 Mean (SD) (*n* = 37)	Posttest T3 Mean (SD) (*n* = 29)	Difference from T1 to T3 Effect Size
Mental component score	43.3 (9.3)	43.9 (10.7)	43.7 (12.1)	0.03
Physical component scores	42.1 (10.1)	44.8 (8.5)	45.7 (9.1)	0.37
Stress	26.5 (13.6)	23.3 (14.3)	24.2 (14.8)^a^	0.16
General self-efficacy	31.2 (3.2)^b^	32.00 (3.8)	32.3 (3.7)^c^	0.32

^a, c^: *n* = 30; ^b^: *n* = 37.

Scores: cancer related stress: 0–75; general self-efficacy: 10–40; mental and physical component scores: mean score: 50. Higher scores indicate better mental and physical component scores, general self-efficacy, and worse cancer related stress.

Abbreviations: T1: test time 1; T2: test time 2; T3: test time 3.

**Table 4:** Adjusted mixed-effect models for longitudinal mean changes in stress, general self-efficacy, and mental and physical component scores (*n* = 37).

Variables	Step 2 explanatory model: stress estimate (SE)	Step 2 explanatory model: general self-efficacy estimate (SE)	Step 2 explanatory model: mental component scores estimate (SE)	Step 2 explanatory model: physical component scores estimate (SE)
Intercept (baseline)	22.9 (9.1)	33.78 (2.56)	43.08 (1.50)	55.08 (5.33)
Change over time	−2.10 (0.78)^b^	0.71 (0.33)^a^	0.36 (0.96)	3.09 (0.91)^b^
Gender (women)	−2.73 (3.96)	−1.17 (1.03)		1.65 (2.87)
Time ∗ gender				−2.77 (1.29)^a^
Age	−0.02 (0.01)	−0.03 (0.64)		− 0.13 (0.08)
Comorbidity (Yes)	1.85 (4.34)	1.06 (1.22)		−8.47 (2.45)^b^
Symptoms (≥1)	7.96 (2.3)^b^	−0.31 (0.64)		−4.86 (1.58)^b^

Age is based on the mean of the sample.

Significance level: ^a^: *P* < 0.05; ^b^: *P* < 0.01.

Abbreviations: SE: standard error of the mean.
